# An in-depth analysis of the representation of speech acts and language functions in Libyan public high school English textbooks

**DOI:** 10.3389/fpsyg.2022.1056745

**Published:** 2023-01-10

**Authors:** Fatma Alhadi Ali Ahmed, Behbood Mohammadzadeh, Farhad Mazlum

**Affiliations:** ^1^ELT Department, Faculty of Education, Cyprus International University, Nicosia, Türkyie; ^2^ELT Department, University of Maragheh, Maragheh, Iran

**Keywords:** textbook evaluation, speech acts, language functions, EFL learners, Libyan context

## Abstract

Researchers have taken an interest to study the language of English materials that learners use in different contexts. One line of inquiry is to examine the pragmatic competence potential of such materials. This study aimed at examining the use of speech acts and language functions in the 21st Century English for Libya Secondary 1, 2, and 3 course books and workbooks. To achieve this aim, a frequency-based analysis of language functions and speech acts in the conversation sections in the five course books and five workbooks was followed. A data analysis revealed that the distribution of speech acts and language functions is highly skewed; in each analysis domain, a given category accounted for almost half of the data while other categories accounted for little or even none. Findings are used to argue that Libyan students need a more balanced and language-in-use informed distribution of English functions and speech acts to learn the pragmatic features of English; otherwise, they are less likely to become communicatively competent in speech acts and functions that have been not been treated adequately. The findings also highlight that the unequal frequency and distribution of these pragmatic variables are in line with previous studies and provide implications for English as a Foreign Language (EFL) curriculum, material designers, and language practitioners.

## Introduction

When learning a language, some competencies are essential for second language (L2) learners to communicate effectively. The lack of these competencies can create obstacles for the learner throughout the learning process. These obstacles are mostly linked to deficiencies in the pragmatic competence of the learners' utterances. Learners who are skilled in the structural and lexical side of the language can also experience these pragmatic deficiencies in their L2 language competence. In most cases, communicative functions in certain contexts are not successfully interpreted, expressed, or delivered (Meihami and Khanlarzadeh, 2015). Pragmatics involves what is understood from an utterance that is produced with the norms and conventions of a certain society or context. Therefore, it is seen to go beyond dictionary meanings of statements. For this reason, pragmatic competence involves dealing with conventions appropriately, which allows speakers to build a conversation that is easily understood or clearly conveyed (Yule, 1996).

The main means by which L2 learners can obtain maximum exposure to pragmatic competence is *via* the textbook. Undoubtedly, textbooks are essential resources in any educational institute. English textbooks in different contexts provide learners with opportunities to learn about the language and its socially and culturally appropriate use (Ahmad and Shah, 2014; Aoumeur and Ziani, 2022). The latter function of textbooks warrants more relevance in English as a Foreign Language (EFL) contexts since their exposure to English is limited compared with English as Second Language (ESL) students (Kim and Hall, 2002; Siswantara and Ariffin, 2021). To see whether English textbooks do what they are expected to do, researchers evaluate them from different perspectives, and one line of inquiry is evaluating whether English textbooks expose students to the pragmatic features of English. In other words, textbooks are evaluated to see whether their use in classes can help learners develop pragmatic competence in English. Alcon and Martinez-Flor (2008) believe that pragmatic competence develops pragmalinguistic and sociopragmatic competence. It is also an advanced skill to comprehend and produce sociopragmatic meanings with pragmalinguistic conventions. Textbooks, therefore, should be evaluated to see whether they have the potential to facilitate the learning of such skills. However, as Diepenbroek and Derwing (2013) highlight, “the quality of the pragmatic information included in textbooks does not allow learners to develop their pragmatic competence in the target language” (p. 1). Many researchers argue that the pedagogical intervention in the teaching of pragmatic competence has a significant impact on learning these skills (House, 1996; Kasper and Rose, 2002; Bacelar da Silva, 2003; Martínez-Flor and Fukuya, 2005; Gu, 2011; Rajabia et al., 2015; Perez-Hernandez, 2021).

The acquisition of sociolinguistic rules is as important as learning the grammatical knowledge of the target language (TL) to communicate effectively (Çakir, 2006). However, it has been demonstrated that, as mentioned previously, even learners who know the structural knowledge of the language well might experience pragmatic flaws. These flaws are often thought to arise from the differences between the native and TL (Thomas, 1995). Indeed, as EFL teachers we experience these flaws from students who have the ability to produce correct utterances in terms of structure but show signs of employing them in inappropriate contexts. An interesting example of pragmatic failure is provided by Roohani and Alipour (2017): “a student who, in response to “Could you help me, please?, says “Yes, I could” instead of “Sure, it would be my pleasure” (p. 26). Therefore, as Moradi et al. (2013) propose, such failures might lead to miscommunications and may also be abided impolite and sometimes even offensive to native speakers.

Seeing the extensive body of information on the pivotal role of pragmatic content in EFL curriculum design, this research sets out to investigate the frequency and distribution of two chief pragmatic features, namely speech acts and language functions in English textbooks taught in Libyan public schools (i.e., 21st Century English for Libya Secondary). Another aim of this study is to observe whether the frequency of speech acts increases with the level of the textbooks.

## Literature review

### The importance of pragmatic competence

Currently, pragmatic competence has received a lot of attention from researchers, especially in the field of language teaching and learning. It is considered to be the key component for developing language competence. Pragmatics involves dealing with language in use and the contexts in which it is used; thus, pragmatic competence is the ability to use language effectively to achieve a specific purpose and to understand language in context. Tulgar (2016) states that “language users are supposed to follow some conventions according to which their conversation will be not only meaningful but also appropriate. This analysis of how to say things in appropriate ways and places is basically called pragmatics” (p. 10). Learning English in our modern world has become noticeably important, thus referred to as the *lingua franca* of the world. Learning a language and learning to use it appropriately and communicatively involves the knowledge of *pragmatic competence*. Unfortunately, the communicative role, in our case pragmatic competence, of language is generally ignored in EFL classes. Instead, most language teachers opt for more traditional methods for teaching English, mainly adopting methods that teach the form (grammar) of language rather than concentrating on how language can be used in authentic and real-life contexts. In Libya where English is treated as a foreign rather than a second language, learners tend to face problems reflecting the production and comprehension of the authentic aspects of the target language, therefore creating hindrances to effective communication (Owen et al., 2019). Therefore, it is natural to see pragmatic flaws more frequently than grammatical ones as Çetinavci and Öztürk (2017).

Nowadays, research on developing competence in English by language learners worldwide is of high significance due to the key role English plays in their lives. One line of inquiry is to examine textbooks learners study because textbooks can help learners develop competence, pragmatic competence included, by exposing them to natural language use which entails speech acts and language functions of different kinds (Taguchi, 2009).

### Textbook evaluation and prior studies

Despite the noticeable advances in technology and the vast number of aids available to enhance any education program, it is a fact that textbooks are still crucial in EFL classes and programs. This is true especially in contexts where textbooks are an indispensable element simply because other pedagogical resources are not available and they are the only available resource.

The evaluation of English Language Teaching (ELT) and EFL textbooks is not a new practice as many language practitioners worldwide have reviewed textbooks from different perspectives such as whether they achieve their stated goals or aims, the design of textbooks, language representation in textbooks, appropriateness of the representation and portrayal of gender, to name a few (Hashemi and Borhani, 2015). All these suggest that the status of the textbook in language classes is still regarded high up on the overall scale and even deemed by some scholars as “the visible heart of any ELT program” (Kirkgoz, 2009). McGrath (2002) states that the evaluation of textbooks is crucial for the administration and development of language learning programs. Accordingly, textbook evaluation is an applied linguistics process whereby language practitioners can “make judgments about the effect of the materials on the people using them” (Tomlinson et al., 2001; p. 15 as cited in Alemi and Irandoost, 2012; p. 200).

Some scholars hold that textbooks used in language classes usually do not seem to allow learners to learn L2 pragmatics, mostly due to not providing frequent opportunities to practice and use speech acts effectively in their natural contexts (Bardovi-Harlig, 1996). For instance, Nevisi and Moghadasi (2020) studied the frequency and appropriateness of speech acts, language functions and politeness markers in Iranian high school textbooks, and the Prospect and Vision Series, as well as investigating the connection between the frequency of the aforementioned pragmatic features and the level of the textbooks. They analyzed 172 conversation excerpts using Searle (1976) speech act theory, Halliday (1978) language function theory, and House and Kasper (1981) politeness structure taxonomy. Their findings suggested that the directive and representative speech acts showed the highest frequency. The informative language function exhibited a higher frequency. However, the committers proved to be the most abundant politeness markers throughout the textbooks. Moreover, the pragmatic features showed an unequal distribution throughout the speech extracts, as well as not showing a significant connection between the frequency of the variables and the level of the textbooks. In a similar vein, in a study conducted by Tran and Yeh (2020), the analysis of the types and distribution of speech acts in Vietnamese EFL textbooks indicated that though there was a presence of speech acts, their distribution and sequence were inadequate. Searle (1976) used Roohani and Alipour (2017) speech act model and Lakoff (1973) model of politeness to study Iranian high school English textbooks. They found that the distribution of speech acts was unequal. Some language functions were even found to show an irregular pattern of presence throughout the books.

Vellenga (2004) compared EFL and ESL textbooks and showed that the textbooks did not enhance acquiring pragmatic competence. She further stressed that the textbooks did not provide enough metapragmatic and metalinguistic information. Despite this, EFL textbooks were richer in coverage given to the pragmatic features than ESL ones. Another study conducted by Takafumi et al. (2007) showed similar results. The researchers evaluated seventeen textbooks used in Japan and approved by the Ministry of Education, Sports, Culture, Science and Technology (MEXT), focusing on the frequency of speech acts in textbooks. The results showed that not all speech acts and their linguistic forms were present. In addition to this, the learners were provided with limited opportunities to practice the forms of speech acts they were introduced to. The metapragmatic information was also found to be lacking “quantity and quality” (Takafumi et al., 2007; p. 8).

On similar grounds, in their study, Siswantara and Ariffin (2021) investigated the conversation sections in a series of Indonesian ELT textbooks to examine whether their pragmatic content is in line with the curriculum expectations. Searle's speech act framework was employed to analyze the illocutionary acts and forces present in the textbooks. The results portrayed that although there was a presence of speech acts, the frequency was quite low and deficient in variety to develop the pragmatic competence of the learners.

The study of Moradi et al. (2013) reveals the unequal distribution of speech acts and language functions in the textbooks they studied. They compared New Interchange Series I, II, and III and Iranian high school English Textbooks I, II, and III. They adopted Searle (1976) speech acts model and Halliday (1978) language functions theory to examine the usage of both speech acts and language functions in the aforementioned series. Their findings revealed that the New Interchange Series had more speech acts, and 1,100 different types of speech acts as opposed to only 225 shown in its counterpart. In addition, the New Interchange Series had more language functions and a more varied selection of these functions than the Iranian high school series. Moreover, the latter set of textbooks presented an unequal distribution of language functions, showing no clear pattern to this distribution. They further pointed out that Iran's locally produced EFL materials cannot help learners develop pragmatic competence. Similar results have been reported by Roohani and Alipour (2017) with regard to Iranian ELT textbooks.

Soozandehfar and Sahragard (2011) analyzed 14 randomly selected conversation excerpts from 14 units in the Top Notch textbooks. Their results showed that the new textbooks at that time were pragmatically insufficient and not functional. Afa et al. (2022) employed Halliday (1978) language function framework to investigate the use of language functions in the conversation sections in the Indonesian seventh-grade textbook *When English Rings the Bell*. The findings were in line with the aforementioned studies as the conversation sections were found to be deficient in some language functions.

The aforementioned review suggests that examining the pragmatic potentials of textbooks is of high significance in different settings. Motivated by this, the present study aims at studying the pragmatic features of EFL textbooks in Libyan public secondary schools.

### Research questions

The study intends to answer the following questions:

**Research Question 1:** What are the types and the frequency of speech acts in the conversation sections in the *21st Century English for Libya* secondary 1, 2 and 3 course books and workbooks?

**Research Question 2:** What kinds of language functions have been used in the secondary 1, 2 and 3 course books and workbooks for 21st century English in Libya and how often?

**Research Question 3:** What does the frequency of speech acts indicate throughout the whole series of the textbooks?

## Method

### Material/corpus

The secondary level of 10 Libyan EFL textbooks, five course books, and five workbooks, by Adrian-Vallance and Gough (2018–2019) encompassed the corpus of the study. The study uses qualitative and quantitative content analysis methods to examine the content of textbooks. The 1^st^ year takes one general course book and workbook. The 2^nd^ year and the 3^rd^ year, however, have two different course books and workbooks, literary sections, and scientific sections, depending on what the learners want to pursue at the university level. All the conversation sections in all the units were the target for the in-depth analysis except for units 4 and 8 of each textbook as they are considered revision units. The reliability of these textbooks has been confirmed by experts in curriculum design. The textbooks are assumed to be designed according to local norms and needs.^1^ In addition, each textbook and workbook for each year and every specialization were regarded as one textbook for easiness and consistency of analysis.

### Framework for data analysis

This study is descriptive in nature; it aims to describe Libyan English textbooks in terms of their use of speech acts and language functions. The analysis of the speech acts and the language functions present in the textbooks involved two main frameworks. The frequency of speech acts was deduced from Searle (1976) speech act theory. Cohen (2006) highlights that the core of all communication in the language is speech acts. It was Austin (1962) who first initiated the speech act theory in his book “How to Do Things With Words”. He stressed that alongside the utterance that a person makes, an action accompanies it. For him, a speech act is the smallest unit or component of communication, for example, making a promise, ordering, or refusing.

In his theory, Austin characterized statements into two types, constative and performatives. Constatives classify statements for either being true or false, like in “It is snowing” or “It is windy today”. On the contrary, performatives do not describe situations but label statements according to a certain performance. Austin stresses that some words in our daily language are not questions or statements but are actions. For instance, when someone confirms he/she agrees to marriage by stating “I do”, he/she in fact emphasizes the action reflected by the verb in the statement when uttering it.

Austin went on to characterize acts or utterances into three categories which are known to be locutionary, illocutionary, and perlocutionary. In simple terms, a locutionary act is the actual verbal meaning of the utterance or the statement, whereas the illocutionary act is associated with the verbal meanings related to particular societies, and the after-effects of an action or an utterance on the hearer is referred to as being the perlocutionary act (El Hiani, 2015). However, the illocutionary act is mostly what the speech act theory revolves around. For example, when someone utters “it is hot today”, this is the locutionary act as it represents the mood of the person or expresses what the person senses or feels. In this state, the illocutionary act deemed by the speaker may involve requesting for opening a window or switching on an air conditioner, or even asking for a cold drink or even something else. Moreover, in most cases, the hearer will act according to the speaker's intentions to carry out the intended perlocutionary act.

Drawing upon Austin's categories, Searle (1976) developed the following five categories: Representatives, mostly known as assertive, are speech acts reflecting actual facts like reporting, stating, or describing, “This is an American car.” Directives promote the hearer to consider encountering the act in future, for example, requesting, commanding, or ordering, “You have pages 1 to 6 homework, have it ready by Tuesday, ok.” The commissive not so different from the prior category requires the speaker to do the action in future, but this time the doer is committed to perform the action, so there is some force involved such as when promising or when refusing, “I'm going to London next week.” In the next category, expressives predict that a person's emotions are greatly involved when completing an action, for example, when congratulating, thanking, complimenting, or apologizing, a person's feelings, state, or attitude is greatly reflected and is mostly considered the whole intention of the proposal, “Thank you for your help and support.” The last category, declarations or declaratives are speech acts that simply connect reality and the proposed content reflected by the actual acts and can lead to changes through sentences or can affect an immediate change of affairs. Such speech acts are acts involving marrying a couple, declaring war, sentencing, dismissing, or even firing from employment, and so on, for example, “You are fired.”

The frequency of the language functions, however, was inferred from Halliday (1978) language function theory. Not long after Searle introduced the modified version of the speech acts, Halliday (1978) introduced the language function theory. In his theory, Halliday stressed that language functions are equally important as speech acts and play a crucial role in pragmatic studies to focus on the communicative intentions of language, which is the aim of language education programs.

Halliday's theory is comprised of the following functions.

1) Instrumental: Language is used to fulfill a desire or need on the part of the speaker. It is the language used to obtain the necessities and comforts of life like food and drink or to satisfy material needs. Also, it can be used for commands, suggestions, and warnings. For example: “I'm hungry, I want a sandwich.”
2) Regulatory: Language is used to influence the actions or behaviors of others like when requesting or when persuading. For example: “Come on,” “Stop it.”
3) Interactional: Language is used for interaction and to develop social relationships. For example, the language used for greetings, leave-takings, apologies, compliments, and insults is interactional. For example: “Sorry,” “Thanks,” “I love you Mummy and Daddy.”
4) Personal: Language is used to express the speaker's preferences, feelings, interests, and actions. For example, “I think Merryland is a good place for families,” “Here I am.”
5) Representational/ Informative: Language is used to request information or exchange information about people and things. For instance, the language used in narrating, identifying, and describing is representational. For example: “I have great news,” “I have something to tell you.”
6) Heuristic: Language is used to learn, explore, detect, or acquire things (i.e., it is used to explore the environment). For example: “What's that,” “Why.”
7) Imaginative: Language is used to imagine, tell stories or jokes, or just create an imaginary environment. The phrase “let's pretend” is a typical example.
8) Attention-Getting Function: Language is used to express many communicative functions, mostly for expressing one's idea, asking questions, giving offers, or typically addressing people. For example “Excuse me.”

The rationale to use two theoretical frameworks to analyze the same materials is to address and examine the *pragmatic load* of Libyan EFL textbooks more comprehensively. According to Bloor and Bloor (1997), Halliday's model of language functions is *functional* and examines how language is used in context, and, complementarily, Searle's speech act theory emphasizes “the social principles of discourse, taking a socio-cultural perspective on language usage, and testing the way that the principles of social behavior are shown” (Poupari and Bagheri, 2013, p. 74). Therefore, the application of the two frameworks is expected to provide a more in-depth picture of our corpus.

The two frameworks were employed to analyze all conversation sections in Libyan EFL textbooks. More specifically, the frequency of each speech act and language function was counted. Every time these features were repeated, they were counted again because the context of use was different. The following extract taken from unit 2 course book 1 is an example of the analysis procedure based on the two frameworks.

*A Sample from 21st Century English for Libya Secondary 1 Course book*.

*Conversation 8 (Course book, Unit 2, Page 39) with analysis based on Searle (**1976**) speech act theory and Halliday (**1978**) language function theory*.

**Woman**: Could you tell me where the bus stop is? [directvie] [heuristic].

**Porter**: Yes. It's around the corner [assertive] [informative]. Just turn right on Bank street and you'll see it [assertive] [informative].

**Woman**: Thank you very much [expressive] [interactional].

After the analysis of the conversation sections, to investigate the distribution of frequency of the speech acts and language functions, the chi-square test was applied.

## Results

**Figure 1:**
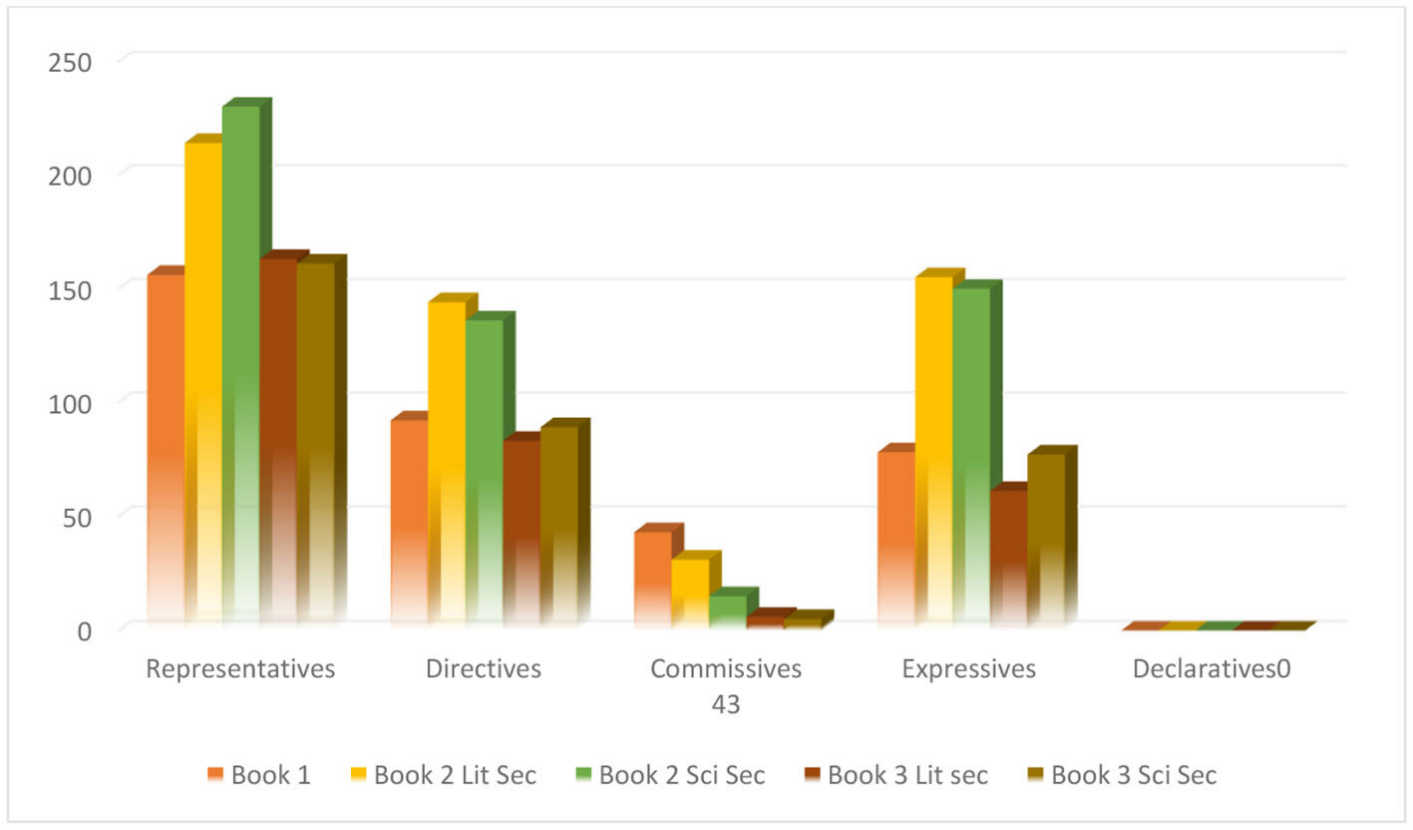
Overall frequency of speech acts in 21st Century English for Libya secondary 1, 2, and 3 course books and workbooks.

The first research question of this research involved the types and overall frequency of the speech acts in the Libyan EFL textbooks. Figure 1 exhibits the types and the frequency of speech acts included in the whole series of all the textbooks. According to Table 1, except for declaratives, all other speech acts are present in the conversation sections of the textbooks, that is, representatives, directives, commissives, and expressives were found in the samples with different frequency rates. The frequencies of the speech acts varied, with the highest frequency rates recorded for representatives in all the textbooks: 156 for book 1, 214 for the literary section of book 2, 230 for the science section of book 2, 163 for the literary section of book 3, and 161 for the science section of book 3. The next mostly found speech act was directives with 92, 144, 136, 83, and 89 cases followed by expressives with 78, 155, 150, 61, and 77 instances in book 1, the literary section of book 2, the scientific section of book 2, the literary section of book 3, and the scientific section of book 3, respectively. Finally, commissives were the least found in the textbooks, starting from book 1 with 43 instances, book 2 (literary section) with 31 cases, book 2 (science section) with 15, book 3 (literary section) with 6, and book 3 (science section) with five commissives. According to Table 2, the results of the chi-square test show that there are significant differences between distribution rates of speech act types [i.e., Asymp. Sig. = 0.000 (*p*-value < 0.05)]. In other words, the main categories of speech acts are not distributed equally.

**Table 1:** Frequency and percentage of speech acts in 21st Century English for Libya secondary 1, 2, and 3 course books and workbooks.

**Speech acts**	**Book 1**		**Book 2 Lit**		**Book 2 Sci**		**Book 3 Lit**		**Book 3 Sci**	
	**Freq**	**%**	**Freq**	**%**	**Freq**	**%**	**Freq**	**%**	**Freq**	**%**
Representatives	156	45.61%	214	39.34%	230	43.31%	163	52.08%	161	48.49%
Directives	92	26.90%	144	26.47%	136	25.61%	83	26.52%	89	26.81%
Commissives	43	12.57%	31	5.7%	15	2.82%	6	1.92%	5	1.51%
Expressives	78	22.81%	155	28.49%	150	28.25%	61	19.49%	77	23.19%
Declaratives	zero	0%	zero	0%	zero	0%	zero	0%	Zero	0%
Total	369	100	544	100	531	100	313	100	332	100

Lit, literary; Sci, science.

**Table 2:** Chi-square results for overall speech acts in 21st Century English for Libya secondary 1, 2, and 3 course books and workbooks.

**Textbook**	**Chi-square**	**Significance**	**Df**
Book 1 (2018–2019)	72.550a	0.000	3
Book 2 Literary Section (2019–2020)	128.926a	0.000	3
Book 2 Scientific Section (2019–2020).	178.009a	0.000	3
Book 3 Literary Section (2017–2018)	162.591a	0.000	3
Book 3 Scientific Section (2017–2018)	147.470a	0.000	3

Figure 1 provides a visual illustration of the findings in this section.

**Figure 2:**
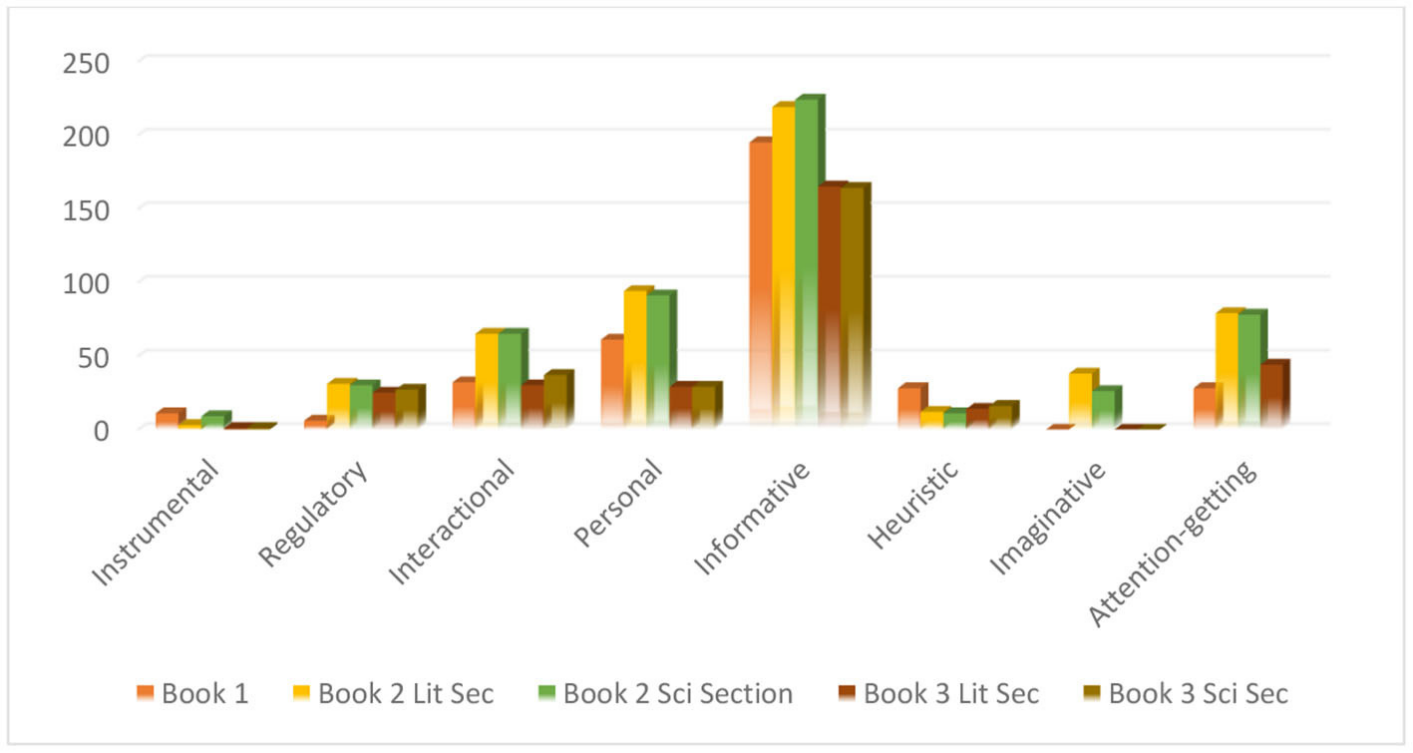
Overall frequency of language functions in 21st Century English for Libya secondary 1, 2, and 3 course books and workbooks.

The second question of this research strived to show the types and frequency of the language functions in all the secondary school EFL textbooks. Frequencies of the pragmatic features pertaining to language functions can be seen in Table 3 and Figure 2 portrays the same information graphically.

**Table 3:** Frequency of language functions in 21st Century English for Libya secondary 1, 2, and 3 course books and workbooks.

**V**	**Book 1**		**Book 2 Lit**		**Book 2 Sci**		**Book 3 Lit**		**Book 3 Sci**	
**Language functions**	**Freq**	**%**	**Freq**	**%**	**Freq**	**%**	**Freq**	**%**	**Freq**	**%**
Instrumental	11	3.04%	3	0.55%	9	1.69%	1	00.32%	1	0.31%
Regulatory	6	1.66%	31	5.73%	30	5.62%	25	08.12%	27	8.44%
Interactional	32	8.86%	65	12.01%	65	12.17%	30	09.74%	37	11.56%
Personal	61	16.90%	94	17.38%	91	17.04%	29	09.42%	29	9.06%
Informative	195	54.01%	219	40.48%	224	41.95%	165	53.57%	164	51.25%
Heuristic	28	7.76%	12	2.22%	11	2.06%	14	04.55%	16	5%
Imaginative	zero	0%	38	7.02%	26	4.87%	Zero	0%	Zero	0%
Attention-getting	28	7.76%	79	14.60%	78	14.61%	44	14.29%	46	14.38%
Total	361	100	541	100	320	100	308	100	308	100

Lit, literary; Sci, science.

According to Table 3, the most frequently used language function is informative, beginning with book 1 that has frequencies of (*f* = 195), (*f* = 219), (*f* = 224), (*f* = 165), and (*f* = 164), from book 1 to book 3. The lowest percentage after imaginative belongs to instrumentals accounting for only (*f* = 11) for book 1, (*f* = 3) for the literary section of book 2, (*f* = 9) for the scientific section of book 2, and *(f* = *1)* for total frequencies of literary and scientific sections of book 3. The next lowest occurrence of language functions was for imaginative with frequencies ranging from none in book 1 (*f* = zero) to (*f* = 38), (*f* = 26), (*f* = zero), and (*f* = zero) in the others. Table 4 gives the frequency information for the other language functions (i.e., personal, attention-getting, interactional, regulatory, and heuristic). According to Table 4, the results of the chi-square test show that there are significant differences between the distributions of language function types and the result is significant at *p-*value < 0.05. The statistical significance of the chi-square result indicates that the main categories of language functions are not distributed evenly and equally.

**Table 4:** Chi-square results for overall speech language functions in 21st Century English for Libya secondary 1, 2, and 3 course books and workbooks.

**Textbook**	**Chi-square**	**Significance**	**Df**
Book 1 (2018–2019)	501.784a	0.000	6
Book 2 Literary Section (2019–2020)	491.473a	0.000	7
Book 2 Scientific Section (2019–2020)	522.839a	0.000	7
Book 3 Literary Section (2017–2018)	413.000a	0.000	6
Book 3 Scientific Section (2017–2018)	384.550a	0.000	6

The third question of this study concentrated on the frequency of the speech acts in accordance with each level. Table 1 and Figure 1 exhibit that there is an obvious fluctuation pattern in the use of speech acts from book 1 to book 3: The frequencies are (*f* = 342), (*f* = 544), (*f* = 531), (*f* = 313), and (*f* = 332). Table 1 shows a noticeable rise in the frequency of representatives and expressives in book 2 for both literary and scientific sections, recording (*f* = 214) and (*f* = 230), and (*f* = 155) and (*f* = 150), respectively, but book one's frequencies of these speech acts appear (*f* = 156) and (*f* = 78) times. This depicts that the frequency of the speech acts rises with the level. It is a different case with directives and commissives because there is a rise in the frequency of the literary sections of book 1 and book 2 from (*f* = 92) to (*f* = 144), and the frequency decreases in the scientific section of book 2 to (*f* = 136). On the other hand, commissives have a rise then decrease pattern, frequencies recording (*f* = 43) for book 1, (*f* = 31) for the literary section of book 2, and (*f* = 15) for the scientific section of book 2. There is no presence of declaratives in the textbooks.

The overall frequency rate of the speech acts decreases in book 3 for both sections; the lowest frequencies of all speech acts have been used in book 3, though the total frequency of speech acts in the scientific section is slightly higher than in the literary section, (*f* = 332) as opposed to (*f* = 313). In general, the results portray that the textbook's level does not necessarily determine the frequency of the speech acts as shown in book 3, and the last stage has the lowest rates.

## Discussion

Since the listening and speaking sections are the target for the specific analysis of pragmatic features, a crucial finding was discovered during this procedure. Unexpectedly, most conversation sections in the textbooks are presented as transcripts and are located at the back of the books, and only book 3 for both literary and scientific sections is situated in the teachers' books which would make it far more difficult for the learners to gain access to the dialogs. Surprisingly, the listening skill, a crucial skill to master, is neglected in the primary, preparatory, and secondary stages of education in Libya (Naeimah Alsuweee Ali, Libyan Ministry of Education teacher trainer, personal communication, September 21, 2020). However, a small percentage of EFL teachers with their own efforts try to portray or depict this skill in the best possible way, which is greatly encouraged by the Libyan secondary school textbook writers, for they explicitly mention in the teachers' book for secondary book 3, literary section that if there are no facilities for using the cassette, teachers can still carry out activities to enhance this skill (p. 7), typically by using different methods and their creativity.

There are 48 conversation sections present as part of the units in all the set of course books and workbooks, and 50 tape scripts at the back of the 1st-year and 2nd-year course books. Only a small percentage of the conversation sections are exhibited in the actual units and also present at the back of the books as tape scripts. The longest conversation section is a tape script 81 lines long, and in comparison, a minimum dialog is 4–6 lines long.

The results of this study reveal that there is an undefined outline of the presence of pragmatic variables within the whole series of textbooks. As evident in Table 1, apart from declaratives, all the other types of speech acts are present in the conversation sections in the textbooks, that is, representatives, directives, commissives, and expressives were found in the samples with different frequency rates. Despite this, the absence of one category of speech acts and, more importantly, the uneven treatment of the others can be considered a drawback in Libyan EFL textbooks since it is important to make learners familiar with all speech acts so that they can communicate effectively. Vellenga (2004) particularly stresses on this point and claims that possessing sufficient or adequate communicative knowledge is essential for interacting, as well as in helping in conveying opinions or viewpoints. In line with Vellenga's view mentioned previously, Searle (1976) highlights that the necessary felicity conditions where all speech acts can be applied must exist. Since after some time the learners' communicative competence usually increases and enables them to form social relationships and shape their beliefs or ideologies, they need to get exposed to all types of speech acts within appropriate examples. So, declaratives being non-existent in the textbooks classify them as having a deficiency. Cutting (2002) states that declaratives are used in our lives and should not be taken for granted. On the contrary, they are vital speech acts in the English language as when they are used, they may change or alter the world. This is recognized in their utterance, such as when saying “I announce,” “I declare,” “I bet,” and “I resign”. So, the lack of exposure to the contexts in which this critical speech act is used will cause deficiencies in both the learners' communicative and pragmatic competencies.

A significant finding in the research which is in line with Roohani and Alipour (2017) and Nevisi and Moghadasi (2020) studies is that the representative and directive speech acts presented the highest frequencies in all Libyan EFL textbooks. The former study recorded 44.40% for representatives and 34.33% for directives for the Prospect 1–3 textbooks and 28.03% for representatives and 43.68% for directives in the Vision 1–3 series in Iran. The latter study, on the contrary, showed 39.59% for representatives and 41.61% for directives. However, a slight difference can be depicted in this study for the results in this study. Table 1 reveals that the expressives for book 2 for both sections had higher percentages than the directive speech act counting for 28.49% for the book 2 literary sections and 28.25% for the scientific section, whereas the directives scored 26.47% for the literary sections and 25.61% for the sci sec. The two speech acts having the highest scores can be based on the crucial but typical assumption that these two speech acts account for most of the content in conversations found in our daily lives, “everyday vocabulary” as Schneider (2022) p. 155, refers to them. Typically, they are found in statements like “no one makes a better cup of tea like mum” when boasting or “could you close the door please” when asking (Roohani and Alipour, 2017, p. 30).

The lowest percentage was recorded by instrumentals accounting for only 1.21% of the total percentage, which is in line with Kohandani et al. (2014) and Farashaiyan et al. (2018) studies, where instrumentals record 8.26 and 8.16%, respectively. The next lowest occurrence with a percentage of 3.10% belongs to imaginatives, which is fairly reasonable even if it is a quite low frequency as its presence is usually rare in other textbooks. Yet, it is only safe to say that this small percentage is located in the conversation sections in book 2 for both the scientific and literary sections with frequency rates of 26 and 38, respectively. The other books have no occurrences of this function. According to Halliday (1978), the imaginative function is very important and effective when communicating in real life, as the language here has a particular manner for reflecting stories and jokes and has a means for creating an imaginary environment. Sheldon (2015) also highlights the importance of the second likely purpose of the imaginative function. This is especially true and important if the objective of the textbooks is to reflect communicative teaching.

However, when comparing the study to other studies regarding the frequency of language functions, Farashaiyan et al. (2018) and Nevisi and Moghadasi (2020) studies were found to be similar in terms of informatives having the highest frequencies in all the set of textbooks that were examined. The former study accounted for 36.48% for the Prospect 1–3 book and 29.23% for the Vision 1–3 book and the latter study 29.75% for the informative language function in Cutting Edge Intermediate textbooks. These results reflect the inquisitive nature of humans when communicating and reveal the importance of this function, which is often and unconsciously taken for granted (Ambrosio et al., 2015).

Overall, the results of the study depicted that like the unequal coverage of the speech acts, the language functions also exhibit an unequal distribution among the series of textbooks, and this is clearly shown in the chi-square (Tables 2, 4). However, when the results of the coverage of the frequency of these features were compared with similar studies conducted in different parts of the world, it was found that all the components of the language functions were included, while other researchers found their study samples lacked the declarative speech act and the imaginative language function (Soozandehfar and Sahragard, 2011; Kohandani et al., 2014; Nourdad et al., 2016; Farashaiyan et al., 2018).

Still, it is considered a significant disadvantage for textbooks to exhibit variability and fluctuation in the distribution of the categories of certain features. Halliday (1975) claims that for a conversation to be good, it must contain all the various parts; in this case, language functions with equal distribution. Although in real-life conversations there may be the presence or absence of some language functions depending on the context or situation, the aforementioned point is particularly stressed upon and must be a condition followed throughout the textbook. A good textbook must contain all the types of language functions evenly and equally distributed to allow maximum exposure and to familiarize the students with the exact usage of the different functions, all solely for benefit of the students. So, even though there is a presence of all the language functions in the textbooks, the textbooks are still considered as having a shortcoming for their uneven distribution and variant levels of frequency. Consequently, in future, material and curriculum designers must consider these abovementioned points, best done by replicating authentic conversation sections or dialogs with the inclusion of all the types of functions with similar frequencies and even distributions (Kohandani et al., 2014).

Moreover, as it might be expected that subsequent levels in education programs should indicate that the components of the materials should be richer in information and knowledge, in this case, speech acts and language functions should be in greater percentages for successful communication to occur. This is not the case for 21st-century English for Libya textbooks as seen in Table 1, which is consistent with Soozandehfar and Sahragard (2011) and Nevisi and Moghadasi (2020) studies exhibiting the uneven sequence of speech acts throughout the levels of the textbooks. It is noticeable that the speech acts for book 3 in English for Libya textbooks in both sections are quite low compared to the other levels because at this stage, the focus is on consolidating information related to the students' academic preference as typically students who wish to enroll in the literary section, their majors at the higher level of education would be mostly in humanities and students who choose to study in the scientific section, and they will obviously enroll in science or medicine later on. Furthermore, it is worth mentioning that the set of books is intended for educational purposes and is not for a general English course. The authors explicitly state that in one of the teachers' books, “[t]his course has been developed for the teaching of English to students in secondary 2” (Adrian-Vallance and Gough, 2016, p. 6). So, books, especially books 2 and 3, as mentioned previously, are intended for certain specializations. The books are full of academic terminology, especially book 3, so most of the available conversation sections and tapescripts' themes, which are mainly located in the teachers' books, are revolved around this.

## Conclusion

To develop the skill to comprehend the veiled meanings in TL utterances is crucial in the language learning process as miscommunication can occur and hearers could be misled into saying something displeasing and provoking if the speaker is not cautious. So by comprehending pragmatics and its various components, like the ones mentioned in this study, one can create a clearer understanding of L2 speech (Hidayat, 2016). The objective of this research was to examine the frequency of two main pragmatic features, namely speech acts and language functions, in 10 Libyan EFL textbooks. The analysis was conducted using two esteemed pragmatic frameworks. The results indicated some shortcomings regarding the frequency occurrence, distribution, and layout of some speech acts and language functions in the whole series of textbooks. This supported Vellenga (2004) study in which she claimed that language learning textbooks, local and global, lacked to show a consistent pattern concerning pragmatic content. This deficiency, unquestionably, will lead to certain obstacles in future, especially problems associated with the level of the communicative performance of the learners in future scenarios. This is mostly due to either the textbooks' focus on some speech acts or language functions and not others. An outstanding finding is that the results seem deceiving in theory as realistically in practice the frequencies are much less due to the position of the conversation sections in the textbooks and the neglect of the listening skill in the EFL classes (Omar, 2013). Therefore, even if some components of a pragmatic feature seem to be enough for the learners to develop a reasonable competence of the TL, the teachers and learners alike do not use the textbooks as planned and advised by the curriculum designers. This will evidently cause drawbacks in the development of the pragmatic competence of the learners.

### Pedagogical implications

The current research suffered from some limitations that should be considered in future studies. First, the study only examined the secondary level investigating two pragmatic features. Thus, more similar studies could be conducted on textbooks at primary, secondary, and tertiary levels to realize the objectives needed to gain good communicative competence. The studies could also include the analysis of other pragmatic features and the strategies that are listed to realize these variables, like the analysis of metapragmatic information. Not forgetting that the textbook is of paramount importance to the teaching and learning process, thus curriculum designers' duty is to make sure that in future teaching and learning material content should be revolved around the development of pragmatic competence. Therefore, material designers should consider adding more authentic representations of the TL. More research should be done on the effect of authentic material on L2 pragmatic development. This would be significantly enhanced if EFL teachers would be integrated more than the textbook in the learning process. The textbook is simply a guide to follow, and the teacher should tailor the books and use the material to cater to his/her learners' needs. As seen from the results section, the books have some shortcomings, so the teacher should play the part of a guide and a facilitator. In simple terms, the teacher, alongside the textbook, should be the heart of the EFL class.

Furthermore, EFL textbooks should be written and revised locally by either highly proficient curriculum designers or even highly qualified EFL teachers. They know what is needed in their local environment and thus can make informed choices and decisions concerning what is necessary for the EFL lessons in the formal classroom setting. It should be as Minh (2017) highlights “these books need to be designed to suit the learners' needs and culture and the contexts of learning and teaching….Textbooks written by native speakers of English…..do not meet these needs (p. 218).”

Another crucial point that needs to be addressed is that material designed for language learning usually provides examples or extracts that are simplified or unauthentic to some extent, which typically does not reflect communication in the real world. The real world is not as straightforward as textbooks portray (Carter, 1998). This is done because if the texts follow a more predictable pattern, it would be more straightforward and effortless for them to follow. They also make texts more straightforward to interpret what has been conveyed simply because they are aware and can predict what expressions may follow. All these may help in the beginning stages of learning a language, but they may face communication difficulties in advanced stages and when facing real-life scenarios (Gilmore, 2004). Many EFL learners experienced this and found that what was introduced to them in class was different in the real world, especially in the countries where the language is spoken (Sawir, 2005).

## Data availability statement

The raw data supporting the conclusions of this article will be made available by the authors, without undue reservation.

## Author contributions

FA: original draft. BM and FM: supervision. All authors contributed to the article and approved the submitted version.

## Footnotes

^1^Ministry of Education, Curricula and Educational Research Centre, 2018–2019.
